# Happy without money: Minimally monetized societies can exhibit high subjective well-being

**DOI:** 10.1371/journal.pone.0244569

**Published:** 2021-01-13

**Authors:** Sara Miñarro, Victoria Reyes-García, Shankar Aswani, Samiya Selim, Christopher P. Barrington-Leigh, Eric D. Galbraith

**Affiliations:** 1 Institute of Environmental Science and Technology (ICTA), Universitat Autònoma de Barcelona, Bellaterra, Spain; 2 Institució Catalana de Recerca i Estudis Avançats (ICREA), Barcelona, Spain; 3 Department of Anthropology and Department of Ichthyology and Fisheries Science (DIFS), Rhodes University, Grahamstown, South Africa; 4 Centre for Sustainable Development, University of Liberal Arts (CSD-ULAB), Dhaka, Bangladesh; 5 Institute for Health and Social Policy, McGill University, Montreal, Quebec, Canada; 6 McGill School of Environment, McGill University, Montreal, Quebec, Canada; 7 Department of Earth and Planetary Sciences, McGill University, Montreal, Quebec, Canada; International Centre for Integrated Mountain Development (ICIMOD), NEPAL

## Abstract

Economic growth is often assumed to improve happiness for people in low income countries, although the association between monetary income and subjective well-being has been a subject of debate. We test this assumption by comparing three different measures of subjective well-being in very low-income communities with different levels of monetization. Contrary to expectations, all three measures of subjective well-being were very high in the least-monetized sites and comparable to those found among citizens of wealthy nations. The reported drivers of happiness shifted with increasing monetization: from enjoying experiential activities in contact with nature at the less monetized sites, to social and economic factors at the more monetized sites. Our results suggest that high levels of subjective well-being can be achieved with minimal monetization, challenging the perception that economic growth will raise life satisfaction among low income populations.

## Introduction

While human well-being is a universal goal of public policy, most metrics used for assessing social progress rely on economic performance [1]. Yet, these metrics fail to capture crucial aspects of social and environmental challenges, such as income inequality, the benefits from informal economic activities or the depletion of natural resources [2, 3]. These omissions can lead to policy choices with unintended negative impacts on people’s welfare and encroachment on planetary boundaries [2–4]. In response to these shortcomings, subjective well-being (SWB), sometimes referred to as happiness, has risen as a promising alternative indicator for societal progress that is more closely aligned with the living conditions that matter to people [3, 5–7]. The quantification of SWB relies on self-reported assessments, and high levels of SWB have been associated with a number of desirable individual and societal outcomes [8, 9]. Given the rising interest, the literature of SWB has expanded dramatically in recent decades.

A wealth of SWB data have been collected from countries across the world [10–12] showing that SWB tends to be quite stable over short periods of time and across regions. Importantly, SWB displays reproducible relationships with objective variables that are consistent across cultures [13–15], providing clues on how changes in personal, social and environmental circumstances affect SWB. These factors can then be used to guide policy decisions in order to raise SWB. Factors that have been associated with SWB include health, age, fulfillment of basic needs, social support and engagement, good governance, civil status, and income [14, 16].

**Fig 1:**
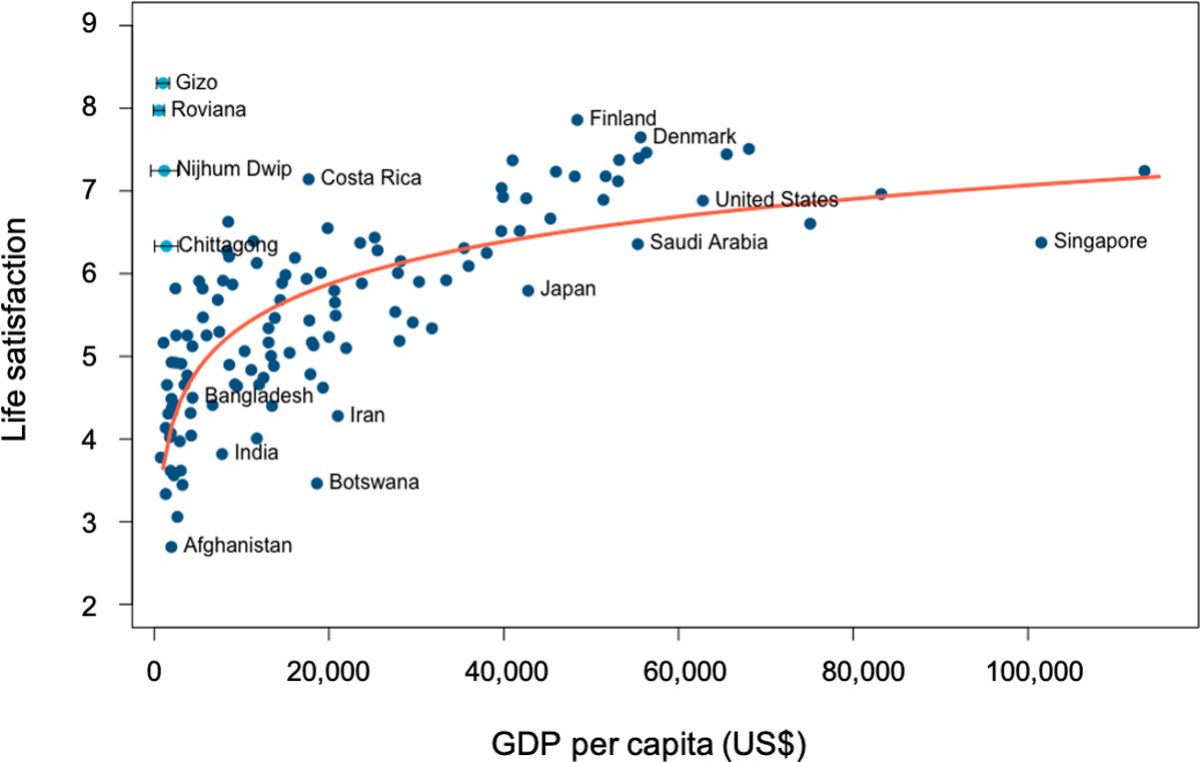
National average GDP per capita (ppp, USD) vs. life evaluations for 2018. Darker dots show mean national responses to the Cantril ladder question [19] against their country’s GDP [20]. In light blue are the mean Satisfaction with Life (SWL) responses from our study sites, represented against their mean local income ± SD (see Methods). The orange line was fit to the Cantril ladder data using a linear regression model and the log of GDP per capita.

Many studies have explored the possible role of monetary income in raising SWB. A consistent trend has been noted between SWB and the logarithm of the per capita gross domestic product (GDP) [17], which implies that the strongest effect of income on SWB occurs among low-income countries (Fig 1). Household income has also been correlated with the life satisfaction of individuals within communities [6, 17], a finding that has been echoed in some low income, non-Western settings [18]. The income-SWB association is consistent with a priori expectations, given that income in monetized societies provides for essential human needs and access to services and amenities.

However, there are reasons to question a fundamental role of monetary income in determining SWB. A large body of literature has explored the observation that many countries do not appear to become happier as they grow richer, a finding known as the Easterlin Paradox [21]. The Easterlin Paradox throws doubt on the strength of the causal relationship between income and SWB. In addition, most of the work on driving factors of SWB has its origins in Western, educated, industrialized, rich and democratic (WEIRD) societies [22], which may not be representative of SWB drivers in other contexts. Furthermore, because income is quantified with money-denominated market exchange values, studies of the income-SWB relationship necessarily exclude non-monetized, subsistence-based societies.

While it can be argued that purely non-monetized societies no longer exist, a number of minimally-monetized societies do persist. In such societies people produce enough to satisfy their own needs, with only minor trade or barter for non-essential goods and services [23]. According to the widespread understanding that income matters more for the SWB of people at low income levels, one would expect that people in minimally-monetized economies would show low SWB. Yet, the fact that happiness is a universal feeling [24, 25] suggests that income may be just a substitute for other sources of happiness, an assumption that is easier to notice in settings where money has little or no use. Here we examine how SWB varies in societies with different degrees of monetization through the use of multiple SWB measures.

We use three independent measures to assess complementary but distinct psychological dimensions of SWB [26]. The three measures were chosen to control against potential issues with regard to cultural differences in understanding the questions, self-reporting, and interviewer bias. The first measure, cognitive life evaluation, is the most widely used aspect of SWB, typically assessed using a single question. This question is phrased in a few different forms, of which we use the Satisfaction with Life question (SWL). Emotional well-being, or affect, refers to the mood resulting from a particular experience and can be considered as the momentary experienced emotional state [27]. The second measure, affect balance, was obtained by asking interviewees what emotions they had experienced throughout the previous day, and calculated as the difference between positive and negative emotions. The third measure, momentary affect, was obtained by querying subjects by telephone at random times about their emotional state.

**Fig 2:**
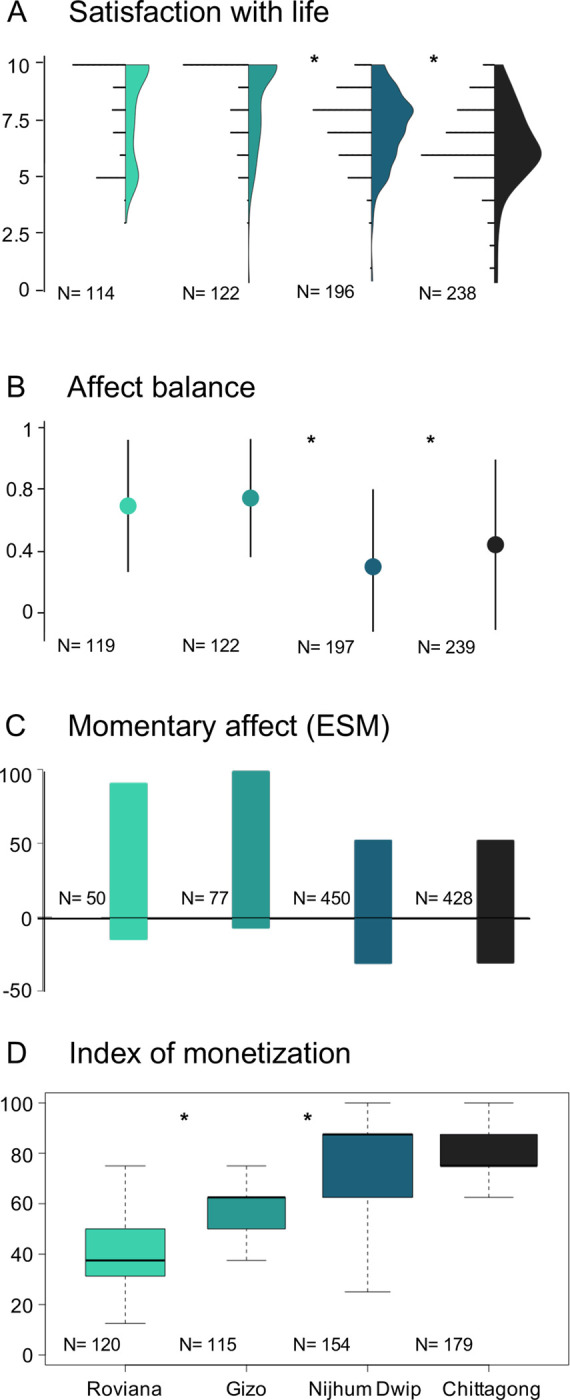
Subjective well-being and index of monetization at the study sites. Satisfaction with life (A) is measured on a 0 to 10 scale. Affect balance (B) is given on a scale from -1 (participants only reported negative emotions) to 1 (participants only reported positive emotions), with vertical lines representing the standard deviation. Momentary affect (C) shows the proportions of positive and negative affect as bars above and below zero, respectively. The index of monetization at each site (D) was calculated from the reported percentages of purchased food and fishing catch sold outside the community. Asterisks indicate significant differences between the sites (S1 and S6 Tables) and the sample size for each metric and site is shown in each panel.

We provide results for coastal communities at four sites in two of the world’s Least Developed Countries, the Solomon Islands and Bangladesh. Small-scale fishing communities like the ones in our study sites are often called "the poorest of the poor" [28]. While income in both countries is very low, the Solomon Islands are more reliant on subsistence activities. The selected sites span a range of cash-dependency, which was estimated by a simple monetization index based on the relative amount of fish sold and food purchased reported by survey participants (Fig 2D and S1 Table).

## Materials and methods

This study was approved by the ethics committee board of the Autonomous University of Barcelona under the reference number CEEAH 4119. All participants were informed of the nature and possible consequences of the study and agreed freely to participate by giving either written or orally recorded consent.

### Study sites

Data collection took place in coastal communities in the Solomon Islands and Bangladesh. In each country, we worked on two sites, one rural and one urban. The sites were selected to exemplify different societal models, cultural values, and to provide substantial variation in the degree of monetization. Both the Solomon Islands and Bangladesh are listed as Least Developed Countries by the UN and strongly rely on small-scale fisheries for their food security and livelihoods. By focusing on small-scale fishing communities, we ensured that all sites have the possibility to have some level of subsistence without money. The selected sites contrast in their level of market integration, i.e. from purely self-sufficient to local trading of fishery products to export. Depending on their number of households, between two and six communities were sampled within each site to cover the sample size targets. Communities within each site were selected for logistical reasons (e.g. we had contacts, communities were willing to collaborate, etc.), while keeping homogeneity in the number of inhabitants and livelihoods and covering the main ethnicities or religious affiliations present in the study sites.

The Solomon Islands are one of the largest Pacific Island states. Around 80% of the population live in rural subsistence communities, while the urban population makes up an estimated 23%, and it has a Human Development Index (HDI) of 0.546 (rank 152 in the world) [29]. The study sites in the Solomon Islands were the Roviana Lagoon (rural site) and Gizo (urban site). In the Roviana region we surveyed the small-scale fishing communities of Bulelavata, Baraulu, Hapai and Olive, in which the land is customary-owned and the sea is governed by customary sea tenure. These communities are ethnically Melanesian and live a subsistence-based lifestyle with infrequent visits to the closest market, a small local marketplace for the region’s peoples to sell their subsistence products. The site in Gizo included the communities of Babanga, Nusa Baruku and Fisheries Village near Gizo Town, all located on Solomon Islands government land. Fishers sell their catch in the daily Gizo fish market, a small regional market for the Western Province and use the money to buy some processed foods or to pay school tuition fees, but their basic needs are in the most part covered through subsistence activities.

Bangladesh is one of the most densely populated countries in the world, 35.9% of it being urban, and has an HDI of 0.608 (world rank 136) [29]. The main ethnicity in Bangladesh is Bengali (98%). The sites in Bangladesh were Nijhum Dwip and Chittagong. Nijhum Dwip, the rural site, is a relatively recent remote island created by a sand alluvium accumulation and colonized by fishermen in the early 1950s. Most fishermen work for a patron, who provides a loan and the boat and fishing gear. Communities depend on money for certain goods and services, particularly food provision, and typically at least one household member engaged in some form of paid work, but household members were involved in subsistence activities. Chittagong, the urban site, is the largest port in Bangladesh and the second largest city in the country. We sampled two communities in the metropolitan area, North Salimpur and Sagorika. While North Salimpur is a small, close-knit permanent Hindu community, nearly half of Sagorika’s workforce is made of immigrants from other parts of Bangladesh, mostly Muslims, coming as temporary laborers. Sagorika, being closer to the city center was more hectic and received a diverse and changing group of buyers. It is also a scenic location within Chittagong commonly visited by weekenders.

### Data collection and analysis

The data collection took place between April and August 2018. Every question in the surveys was designed to be as unambiguous and transferable across languages as possible, trying to reduce the possible biases and heuristics that may occur among different cultural settings [30]. A pilot study was conducted in the Solomon Islands to test the questionnaires suitability and length, and necessary adjustments in the phrasing of questions were made. At each site, three data collection methods were used: (1) structured interviews to obtain information of participants’ subjective well-being, fishing activities and lifestyle, (2) phone interviews with fishers to assess their momentary affect, and (3) a free-listing exercise of what participants considered makes them happy. Each of the three methods had a different sampling strategy and data analysis, which are detailed below. All the statistical analyses and graphs were performed using R, version 3.5.2. Results are presented as mean ± standard deviation (SD) throughout the manuscript and considered statistically significant when p < 0.05.

#### Satisfaction with Life (SWL), affect balance, local income and monetization index

SWL, affect balance and the data used to calculate the local income and construct the monetization index were collected via structured interviews (N = 678). Since the logistic limitations associated with the remote sites made conducting a pilot study to estimate variation in answers unfeasible, the minimum sample size per site was established at 120 [31]. Participants were selected by random sampling from a household list of residents obtained from community leaders in the Solomon Islands, and by convenience sampling in Bangladesh due to the larger village sizes and lack of complete household registries. To reduce sampling error, communities in Bangladesh were spatially divided in a grid and convenience sampling was performed by enumerators in randomly selected areas within the grid. Mean age of participants was 37.2 ± 13.8, from which 567 (83.6%) were male and 111 (16.4%) were female. This gender asymmetry was due to difficulty in finding female participants, especially in Bangladesh where the proportion of female participants was only 8.5%. Despite our efforts to overcome the inherent logistic hurdles, the external validity of our findings is likely lower for the study sites in Bangladesh.

The structured interviews lasted between 30 and 45 minutes and were designed to collect data about participants' fishing practices, sociodemographic factors, and connectedness with the global market. Participants were asked to rate their life satisfaction (which was asked as the first question of the survey to avoid biasing the participants), and respond to affect questions about the previous day, i.e. how they felt with regard to a selection of positive and negative emotions [32].

Of the standard life evaluation questions, SWL was employed because it uses the simplest and most straight-forward wording [30, 33]. SWL is measured on a 11-point scale and is the measure adopted by the World Values Survey [11]. Another life evaluation method, used by the Gallup World Poll, is the Cantril ladder question. The Cantril ladder, also measured on a 11-point scale, has been shown to produce slightly lower scores than the SWL; however, the two measures display a very high correlation (R = 0.94) when asked to the same people, and were found to produce essentially identical country rankings and very similar correlates, including the effect of income [30]. After pilot testing in the Solomon Islands, it was determined that the SWL question was easier to understand to participants. SWL data were used as reported by participants.

Prior studies have shown that affect balance shares variance with both life satisfaction and disaggregated measures of emotional experience at particular times [34]. Affect can be measured by asking research subjects whether they experienced each of six different positive and negative emotions during the previous day; negative emotions are then subtracted from positive to obtain their “affect balance” [35]. Affect balance was calculated as the average of three positive and three negative emotions: sadness, anger, and worry were subtracted to happiness, enjoyment, and smiling or laughing, producing an index ranging from -1 (completely negative affect balance) to 1 (completely positive affect balance).

To calculate the local income at each site, we asked participants to estimate their household’s monthly income bracket by offering a list of typical income ranges in each country. The mean value of each range was then converted to USD, divided by the number of household members (estimated using the marital status and number of children in the household), and scaled to a yearly income per capita. The values represented in Fig 1 correspond to the mean ± standard deviation for each site.

Interviews included two questions to estimate the degree of monetization, as defined in the manuscript. We asked participants to estimate: (1) the percentage of catch (the main livelihood activity) typically sold outside their community and (2) the percentage of food typically purchased from a store (as opposed to food produced by themselves). We then calculated the mean between these percentages for each participant and represented them in a boxplot (Fig 2D). Our index is a simplification of the complex process of societal monetization and may omit important aspects of this transition. For instance, rural Nijhum Dwip’s dependence on purchased food is overemphasized while its lack of access to large markets where people can acquire other goods and services is missing. Therefore, the index is merely used to illustrate the range of cash dependency of our sites. S1 Table shows the summary statistics for our monetization index for each site, which was significantly different among the sites (p < 10^−16^).

Two-sided Kruskal-Wallis tests were used to assess the differences in SWL, affect balance and monetization index between the sites. These data can be found in the S1 Data.

#### Momentary affect

Participants' momentary affect was assessed via the Experience Sampling Method (ESM) [36]. This method provides direct assessments by querying research subjects at random times throughout the day. By prompting participants to answer questions about their current mood and activities, this measure avoids potential recall bias, and identifies subjective experiences that complement the emotions captured by the affect balance measure.

Voluntary fishers (N = 77) were recruited as participants and either lent a mobile phone (in the Solomon Islands, as the majority of participants did not own one) or asked for their mobile phone number (in Bangladesh), so they could respond to calls at all times. Participants in the Solomon Islands belonged to the communities of Ha’apai (in Roviana) and Nusa Baruku (in Gizo), and were called twice a day for one week in April and May 2018, respectively. Participants from Bangladesh belonged to the communities of Namaar Bazaar (in Nijhum Dwip), North Salimpur and Sagorika (in Chittagong). They were called twice a day during two weeks in separate months (July and August 2018). A summary of the ESM sample is shown in S2 Table; because of the intensive sampling technique and to adhere to anonymity ethical requirements, only the gender and age of respondents was recorded. Participants were called once in the morning and once in the evening, at random times and responded to a quick phone interview including questions about what they were doing at the moment and what emotions they were feeling. Total recorded phone interviews were N = 1002 in Bangladesh, and N = 163 in the Solomons. The lack of infrastructure (e.g., access to electricity) in the Solomon Islands meant that participants’ phones often ran out of battery and response rates were low, thus producing a significantly lower sample size. Conversely, the study could be reproduced twice in consecutive months in Bangladesh. To record participants’ momentary affect, they were offered a list of positive and negative emotions, and physical states, and they could choose as many as they felt fit their current mood. This list was prepared and then tested and refined in a pilot study in Roviana (Solomon Islands). The original data can be downloaded from the S2 Data.

The emotions reported in each call were characterized as positive and/or negative. Positive emotions included “happy”, “well”, “smiling”, and “satisfied”. Negative emotions included “worried”, “anxious”, “not good at all”, “angry”, “scared”. If participants reported only physical problems or needs, such as “tired” or “hungry”, as opposed to clearly positive or negative emotions, or if they did not report any emotions, the call was excluded from the analysis. Positive or negative emotions were processed separately as presence/absence data, so that the same call could report both positive and negative emotions, although that was uncommon. The percentage of positive and negative responses was calculated for each site and are reported in Fig 2C and S3 Table.

#### Robustness of SWB results

Robustness tests were performed to check for cultural biases in SWL responses (S4 Table). Cultures with low numeracy have been reported to simplify the available scale and limit their responses to central and extreme options [37]. To test whether this effect could have modified our results, SWL answers were aggregated in a 3-point scale containing answers 0–3, 4–7, 8–10 and the same summary statistics were applied to check for consistency in the results. For the affect balance robustness test, we reproduced the Kruskal-Wallis tests with the average of the affect balance with the full set of questions that were originally asked about yesterday, a total of 7 pertaining to positive affect, and 4 pertaining to negative affective states.

#### Differences in perceived drivers of happiness among sites

**Fig 3:**
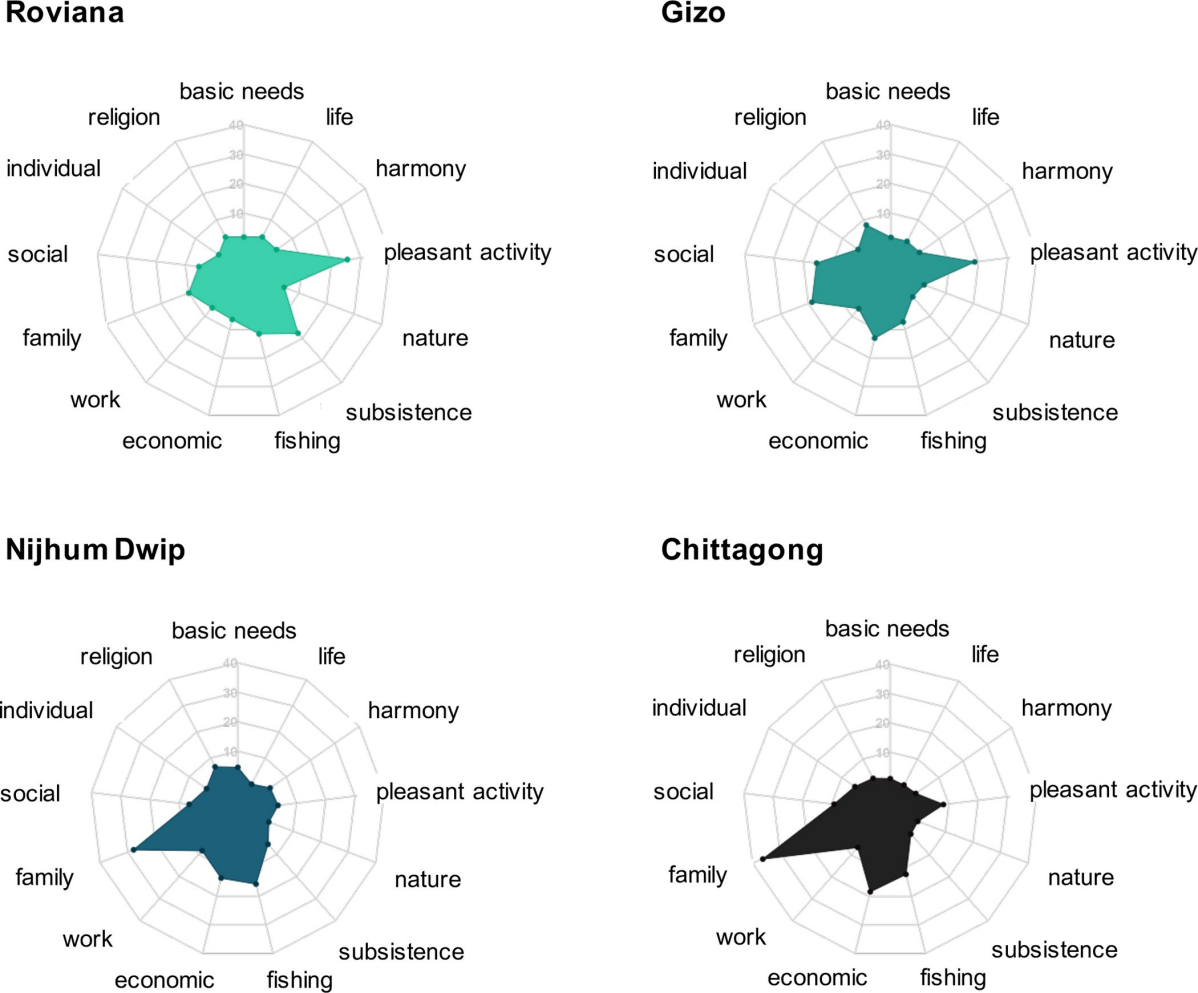
Reported drivers of SWB at the study sites. The distribution of all answers is shown for each site, as classified among inductive categories determined by peer coding.

To assess qualitative differences in the perceived drivers of subjective well-being, a total of 348 participants (between 66 and 113 in each study site) were selected by convenience sampling and prompted to list the three main things that make them happy. Their responses were then categorized based on similarity of concepts by peer coding [38], in which two researchers classified all responses independently and then compared their results until consensus was reached. Some responses could have been classified in more than one category, and consensus was reached during the peer coding exercise as to how to prioritize the classification of conflicting answers. For instance, an answer such as “delicious meals with my family” which could be classified either as “family” or as “pleasant activity”, was decided to go into the “family” category. Answers related to romantic love, including “my husband”, “seeing my wife’s smile”, “kissing my girlfriend” were categorized as love in the “individual” category as opposed to “family”, which included responses such as “my husband and my children”, or “seeing my family happy”. Fishing appeared frequently as it permeates many aspects of daily life in small-scale fishing communities. In the communities studied here, people engage in fishing both as subsistence and income generating activity, which could not be distinguished from their responses. Thus, if fishing was clearly referred to as an economic means (e.g. “catching lots of fish to get a high income”, “high income from fishing”, etc.), the response was categorized as economic; otherwise, it was coded as subsistence/fishing. The original responses given by participants (items 1–3) and our peer coding classification (codes 1–3) can be found in S3 Data. The percentage of total responses was calculated and represented in Fig 3 of the manuscript.

**Fig 4:**
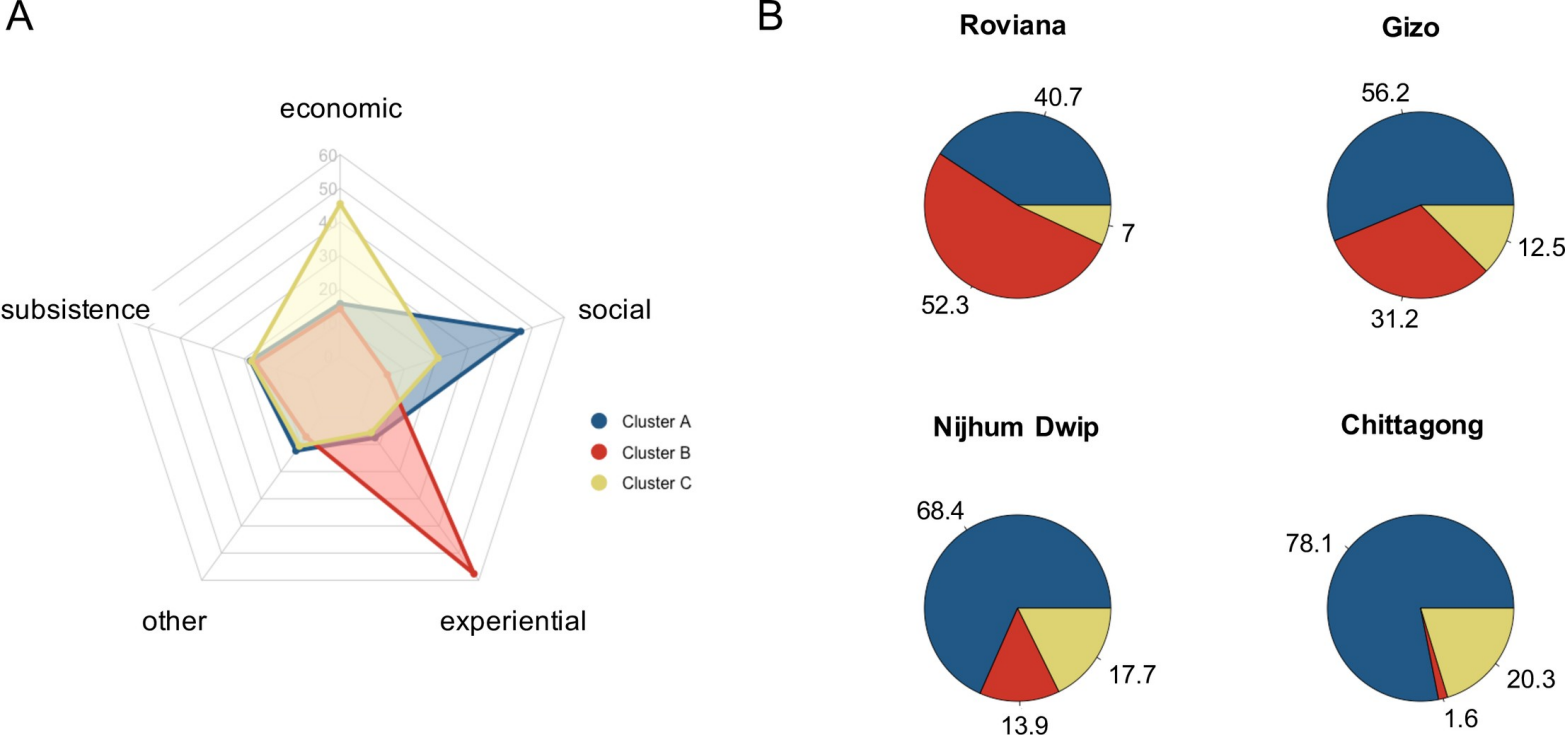
Cluster analysis from perceived SWB drivers. The radar plot (A) displays the three clusters based on the grouping of the responses shown in Fig 3. (B) shows the cluster distribution by site as the percentage of respondents that fall within each cluster.

The responses were further aggregated into 5 broad categories—social, experiential, economic, subsistence/fishing, and other- to allow for unstructured clustering of the responses. S5 Table shows representative examples of responses classified in each category. We used the k-modes algorithm [39] to cluster response categories based on how frequently they appeared together in participants’ answers. Three distinct clusters were obtained, and their frequency of appearance in each study site was calculated and is shown in Fig 4.

## Results and discussion

### High subjective well-being with minimal monetization

Despite the complex nature of SWB, the three measures showed a decreasing trend with increasing monetization (Fig 2). Mean SWL generally decreased from Roviana to Chittagong, which had the lowest SWL (Fig 2A and S3 Table) and the highest index of monetization. Mean SWL in Roviana and Gizo, the less monetized sites, was not significantly different but very high (8.14 ± 2.11, S6 Table). Affect balance showed high dispersion but was also higher in the Solomon Islands (0.71 ± 0.41) than in Bangladesh (0.36 ± 0.54), did not differ between Roviana and Gizo, and was different between Nijhum Dwip and Chittagong. Nijhum Dwip had the lowest overall affect balance, the only discrepant measure in the study. Since affect tends to show higher sensitivity to short-term circumstances, we attribute this discrepancy to the frequent hurdles reported by participants in Nijhum Dwip, including exposure to extreme weather events, lack of hospital infrastructure, and frequent harassment by pirates [40]. Momentary affect was remarkably similar between sites in the same country. Nearly all ESM responses in the Solomon Islands (N = 163) reported positive emotional states while only < 10% reported negative emotional states (Fig 2C). In Bangladesh, 52% of responses (N = 1075) reported positive emotional states and 31% reported negative emotional states (S3 Table). Thus, despite the inherent variability in primary data, all three measures show the highest levels of SWB at the least monetized sites.

Fig 1 illustrates the degree to which our results diverge from the expectation that happiness requires high incomes, focusing on SWL, the SWB metric that has been most strongly associated with national GDP per capita. In the Solomon Islands, where 80% of the population is self-sufficient, our mean SWL of 8.1 was consistent with a previous study in the country (SWL = 7.30 ± 2.33 for Solomon Islands and Vanuatu sites combined) [41] and half the respondents reported SWL ≥ 9 and an affect balance of 1 (meaning they only reported positive emotions). These values do not differ substantially from that of Finland, which had the highest reported national average during our study year at 7.9 (using the Cantril’s ladder question) [11], or Denmark, which had the highest national average SWL historically reported at 8.2 [22].

For Bangladesh, SWL at our study sites was higher than the reported national average for the same year (i.e. 4.5 in 2018) [10]. While this discrepancy could be partly caused by cultural sensitivity to the question phrasing (we used SWL while the national average is based on Cantril’s ladder), it could also be that inhabitants of small coastal communities are happier than the average Bangladeshi citizen as captured in the Gallup survey. This possibility would be consistent with our finding that SWL in rural Nijhum Dwip was significantly higher than in the more urbanized Chittagong sites, while the latter are closer to the expected GDP-life satisfaction curve (Fig 1).

A potential confounding variable that is often associated with market integration is access to technology and communication networks. Access to information from faraway cultures with different lifestyles may change the standards people have for a good life and raise the standard against which they compare their living conditions, all of which may be reflected in their SWB [14]. However, our SWB measures do not appear to show a strong bias with access to technology. For instance, SWB at the urban site in the Solomon Islands, which is better connected, was not significantly different from the rural and more remote site, and affect balance in Nijhum Dwip was lower than Chittagong, which is greatly globalized in terms of communication. Thus, although access to technology and information is a recognized factor in people’s life evaluation, it does not obviously exceed other influences in our study.

Despite the complication of within-country variation, the pattern between the study sites holds and is consistent across the three independent SWB measures: SWB decreases with the degree of monetization in our study sites. Thus, not only may income be insufficient to measure what matters for well-being [1, 3], but the relationship can be reversed when applied to lesser-monetized societies.

### Sources of happiness

To further investigate the observed discrepancy with a priori expectations, we analyzed the factors identified by respondents to be important for their happiness in each of the study sites. To avoid imposing cultural biases in the conceptualization of happiness [23], we used a free-listing exercise in which participants (N = 348) were asked to rank the three main factors they considered to make them happy.

As with the SWB measures, the most frequently reported factors showed consistent differences between study sites (Fig 3). Pleasant activities such as listening to music, relaxing, or going for a walk by the seaside were frequently cited at the less-monetized sites, and the frequency of these factors decreased with monetization. Family factors, such as seeing parents happy or spending time with relatives, were common at all sites, but their frequency increased markedly with increasing monetization, as did the frequency of answers related to economic aspects, such as having a high income or selling their fishing catch. Fishing and subsistence activities were more important in the rural sites, while social factors, like playing games with friends or going to parties, were more common at urban sites.

To more clearly identify changes along the monetization gradient, we carried out a clustering analysis of the co-occurring happiness factors mentioned by participants, which revealed three main clusters (Fig 4A). Cluster A is dominated by answers related to family and social activities. Cluster B is characterized by experiential answers, which contain reasons such as pleasant activities, love, harmony, nature and participants’ lifestyle. Cluster C is dominated by answers related to economic activities or outcomes, such as having a high income.

The cluster analysis indicates a consistent trend in the perceived drivers of SWB along the gradient of increasing monetization. More than half of the answers at the least monetized site were contained in Cluster B, reflecting simple pleasures and contact with nature, but the frequency of this cluster decreased steadily with monetization (Fig 4B). In contrast, the more monetized sites were dominated by answers belonging to Cluster A and Cluster C, reflecting social and economic factors. This shift could reflect cultural differences between the less monetized Solomon Islands and more monetized Bangladesh, but the observed trend is consistent with the monetization gradient. Although monetization itself is unlikely to be the only driver of this trend, we hypothesize that it may reflect a tendency for how perceptions of happiness change when societies transition from subsistence to monetized economies.

At subsistence sites, people live in close contact with nature in their everyday lives, often embedded within biodiverse ecosystems. Recent work has suggested a universal human tendency to obtain well-being benefits by spending time in natural environments [42–44], with a stronger effect in pristine and biodiverse environments [45], and by engaging in physical activity in nature [46]. The prevalence of Cluster B at the subsistence sites is therefore aligned with the provision of well-being benefits from the natural environment.

Having a supportive and cohesive social network, or high social capital, is also widely recognized as a universal driver of SWB [16]. It might therefore appear curious that the frequency of social factors was low at subsistence sites, almost doubling across our gradient of monetization. However, because our question asked for perceived drivers of happiness, respondents were unlikely to report factors for which they had not experienced a large variation. Thus, if respondents rarely felt a significant lack of social support, they would be less likely to list social factors as important sources of happiness. Consistent with this, traditional subsistence practices have been shown to increase kin and kith solidarity as the entire community contributes to these activities, and connect contemporary communities to cultural traditions and their elders [47]. In contrast, with increased monetization people often spend more time working, away from their close relatives, and engage in uncertain social interactions with strangers rather than in their small, close-knit subsistence communities [48, 49]. In the same way, economic factors may barely appear in the lesser-monetized site because people do not depend on money to fulfil their basic needs, and money does not play a central role in determining self-worth. The identified happiness drivers are therefore likely to reflect both fulfillment of perceived deficiencies and enriching experiences, dependent on learned expectations.

## Conclusions

Our findings from minimally monetized societies challenge the prevailing view that economic growth is a reliable pathway to increase subjective well-being. While the data presented here were collected only in two countries, and must therefore be extrapolated with caution, this is the first study to our knowledge that systematically compares standardized SWB measures in minimally-monetized, very low-income societies. Culture plays a major role on how happiness is perceived and conceptualized [15, 23], and likely has complex influences on our measures of SWB. Nevertheless, among the populations studied here, our findings are highly consistent across the three independent SWB metrics, which are unlikely to share the same cultural effects or measurement problems. The highest SWL occurred with the least degree of monetization and was comparable to the SWL reported from high income countries. The results provide an unusually clear substantiation of the often-discussed importance of non-material and non-market determinants of SWB and raises reasonable doubts to the income-happiness debate as a whole, suggesting that this debate could be missing a part of the picture when it comes to subjective well-being.

Economic growth and development are often perceived as an essential step for improving human welfare in developing societies [50], but our findings suggest that high subjective well-being, an important component of this equation, can also be achieved by focusing directly on the drivers of SWB, such as provision of basic needs, access to healthy natural environments, and social cohesion. Our findings also overturn the view, based on the income-happiness association, that sustainability is incompatible with high levels of happiness [51], since they prove that very high SWB can be achieved in self-sufficient societies with low material impact. By providing a perspective far removed from the industrialized world, minimally-monetized societies may hold essential insights on the fundamental drivers of human happiness.

## Supporting information

S1 Table Summary statistics of monetization index and results from significance tests between the study sites. (DOCX)

S2 Table ESM participants characteristics by site. (DOCX)

S3 Table Summary statistics of subjective well-being measures stratified by site. SWB measures are given as mean ± standard deviation. The Kruskall-Wallis tests were run including all the study sites.
(DOCX)

S4 Table Summary statistics of the robustness tests for subjective well-being measures stratified by site. SWB measures are given as mean ± standard deviation. The tests included all sites. The robustness test for SWL, consisting on simplifying the scale by aggregating the responses to a central value, yielded the same patterns with sites as the full scale SWL value. For affect balance, results were slightly lower than the standard affect balance, but the same pattern was consistent across sites.
(DOCX)

S5 Table Categories used in the analysis of differences in happiness definitions. (DOCX)

S6 Table Results from the Kruskall-Wallis tests comparing SWL and affect balance between the study sites. (DOCX)

S1 Data Satisfaction with Life (SWL), affect balance, local income and monetization index. This file contains the raw data collected for SWL and for the calculation of affect balance, local income, and index of monetization, along with the date, location and site for each respondent. Yes (1) or no (0) responses to affect questions about the day before were used to calculate the “affect recall index” (using all positive and negative emotions asked to participants), affect balance, positive affect and negative affect.
(XLSX)

S2 Data Momentary affect data. This file contains the raw data from phone interviews, along with participant ID, date and time of the call, and location of respondents. Ha’apai was the village sampled for Roviana, Nusa Baruqu for Gizo, and ND refers to Nijhum Dwip.
(XLSX)

S3 Data Happiness factors. This file contains the original responses (items 1–3) given by participants to the question “What makes you happy?”, as well as the peer coding classification (codes 1–3).
(XLSX)

S1 File This file contains an interview guide containing the questions asked to obtain the data uses as a basis for this study. (DOCX)
