# Supplementary material for: Happy without money: Minimally monetized societies can exhibit high subjective well-being
Source: PLoS One. 2021 Jan 13;16(1):e0244569. doi: 10.1371/journal.pone.0244569 (PMC7806144; doi:10.1371/journal.pone.0244569)
Supplement: S1 Table — (DOCX) [file pone.0244569.s001.docx]

S1 Table. Summary statistics of monetization index and results from significance tests between the study sites.

| **Site** | **Monetization index (mean ± SD)** | **Range (min, max)** | **N** | **Chi-square** | **p** |
| --- | --- | --- | --- | --- | --- |
| Roviana | 42.81 ± 16.42 | (0, 87.5) | 120 | 18.419  56.239  1.742 | 1.77·10^-05^  6.42·10^-14^  0.187 |
| Gizo | 56.70 ± 14.07 | (25, 87.5) | 115 |  |  |
| Nijhum Dwip | 81.14 ± 15.27 | (25, 100) | 154 |  |  |
| Chittagong | 76.34 ± 18.71 | (37.5, 100) | 179 |  |  |
