# Supplementary material for: Happy without money: Minimally monetized societies can exhibit high subjective well-being
Source: PLoS One. 2021 Jan 13;16(1):e0244569. doi: 10.1371/journal.pone.0244569 (PMC7806144; doi:10.1371/journal.pone.0244569)
Supplement: S2 Table — (DOCX) [file pone.0244569.s002.docx]

S2 Table. ESM participants characteristics by site.

| **Location** | **N** | **% male** | **Age** |
| --- | --- | --- | --- |
| **Roviana** | 17 | 59 | 40.7 ± 14.3 |
| **Gizo** | 13 | 92 | 40.4 ± 14.6 |
| **Nijhum Dwip** | 27 | 100 | 25.1 ± 4.7 |
| **Chittagong** | 20 | 100 | 31.7 ± 7.7 |
