# Supplementary material for: Happy without money: Minimally monetized societies can exhibit high subjective well-being
Source: PLoS One. 2021 Jan 13;16(1):e0244569. doi: 10.1371/journal.pone.0244569 (PMC7806144; doi:10.1371/journal.pone.0244569)
Supplement: S3 Table — SWB measures are given as mean ± standard deviation. The Kruskall-Wallis tests were run including all the study sites. (DOCX) [file pone.0244569.s003.docx]

S3 Table. Summary statistics of subjective well-being measures stratified by site. SWB measures are given as mean ± standard deviation. The Kruskall-Wallis tests were run including all the study sites.

| **Subjective well-being metric** | **Assessment methodolgy** | **Solomon Islands** | | **Bangladesh** | | **Chi-square** | **p** |
| --- | --- | --- | --- | --- | --- | --- | --- |
|  |  | **Roviana** | **Gizo** | **Nijhum Dwip** | **Chittagong** |  |  |
| **SWL (0-10)** | Structured interview | 7.97 ± 2.11  (N= 114) | 8.30 ± 2.10  (N= 122) | 7.24 ± 1.77  (N= 196) | 6.33 ± 2.0  (N= 238) | 95.882 | <10^-16^ |
| **Yesterday’s affect balance**  **(-1,1)** | Structured interview | 0.68 ± 0.43  (N= 119) | 0.73 ± 0.39  (N= 122) | 0.28 ± 0.51  (N= 197) | 0.43 ± 0.56  (N= 239) | 97.011 | <10^-16^ |
| **Momentary positive affect (ESM)** | ESM | 91.8%  (N=50) | 100%  (N=77) | 52.7%  (N= 450) | 52.6%  (N= 428) |  |  |
| **Momentary negative affect (ESM)** | ESM | 14.3%  (N=49) | 6.5%  (N=77) | 30.7%  (N= 450) | 30.4%  (N= 428) |  |  |
