# Supplementary material for: Happy without money: Minimally monetized societies can exhibit high subjective well-being
Source: PLoS One. 2021 Jan 13;16(1):e0244569. doi: 10.1371/journal.pone.0244569 (PMC7806144; doi:10.1371/journal.pone.0244569)
Supplement: S4 Table — SWB measures are given as mean ± standard deviation. The tests included all sites. The robustness test for SWL, consisting on simplifying the scale by aggregating the responses to a central value, yielded the same patterns with sites as the full scale SWL value. For affect balance, results were slightly lower than the standard affect balance, but the same pattern was consistent across sites. (DOCX) [file pone.0244569.s004.docx]

**S4 Table. Summary statistics of the robustness tests for subjective well-being measures stratified by site.** SWB measures are given as mean ± standard deviation. The tests included all sites. The robustness test for SWL, consisting on simplifying the scale by aggregating the responses to a central value, yielded the same patterns with sites as the full scale SWL value. For affect balance, results were slightly lower than the standard affect balance, but the same pattern was consistent across sites.

| **Site** | **Roviana** | **Gizo** | **Nijhum Dwip** | **Chittagong** | **Chi-square** | **p-value** |
| --- | --- | --- | --- | --- | --- | --- |
| **SWL (0-2)^1^** | 1.58 ± 0.52  (N= 80) | 1.68 ± 0.54  (N= 81) | 1.14 ± 0.51  (N= 74) | 0.94 ± 0.45  (N= 140) | 116.17 | <2.2·10^-16^ |
| **Global yesterday’s affect balance^2^**  **(-1,1)** | 0.62 ± 0.39  (N= 119) | 0.69 ± 0.36  (N= 122) | 0.23 ± 0.43  (N= 197) | 0.35 ± 0.49  (N= 239) | 127.42 | <2.2·10^-16^ |

^1^ SWL data were aggregated in 3 categories from 0-2 to account for possible cultural bias in participant responses: 0 includes responses from 0-3, 1 includes responses from 4-6 and 2 includes responses 7-10.

^2^ Global yesterday’s affect used for the robustness test including all the questions, including: whether respondents experienced anger, enjoyment, happiness, sadness, stress, or worry; did something interesting, were treated with respect, smiled or laughed a lot; felt healthy; and would like to have more days like yesterday.
