# Supplementary material for: Happy without money: Minimally monetized societies can exhibit high subjective well-being
Source: PLoS One. 2021 Jan 13;16(1):e0244569. doi: 10.1371/journal.pone.0244569 (PMC7806144; doi:10.1371/journal.pone.0244569)
Supplement: S5 Table — (DOCX) [file pone.0244569.s005.docx]

S5 Table. Categories used in the analysis of differences in happiness definitions.

| **Cluster categories** | **Peer coding categories** | **Response examples** |
| --- | --- | --- |
| **Social** | Social | *“helping the poor”, “playing cards with friends”, “make friends”, “going to parties”* |
|  | Family | *“family meals”, “see parents happy”, “spending time with my family”* |
| **Experiential** | Life | *“stay at home”, “living in this world”, “being alive”* |
|  | Harmony | *“peaceful environment”, “good living”, “seeing happy people around me”* |
|  | Pleasant activity | *“drinking tea”, “eating nice food”, “singing”, “nightfall”, “playing”, “sleeping”* |
|  | Nature | *“flowers”, “swimming in the sea”, “picnic”, “seeing ducklings”* |
| **Economic** | Economic | *“high income”, “shopping”, “selling fish at the market”* |
|  | Work | *“improving my business”, “getting better job”, “building houses”, “trading peacefully”* |
| **Subsistence livelihood** | Fishing | *“fishing”, “touching and repairing nets”, “diving for fish every day”, “catching lots of fish”* |
|  | Subsistence | *“gardening”, “cultivating crops”,* |
| **Other** | Basic needs | *“having a land to live”, “having a house”* |
|  | Religion | *“prayer”, “travelling to Mecca”, “attending church activities”* |
|  | Individual | *“having success”, “my husband”, “love”, “managing by myself”, “being respected by people”* |
