# Supplementary material for: Happy without money: Minimally monetized societies can exhibit high subjective well-being
Source: PLoS One. 2021 Jan 13;16(1):e0244569. doi: 10.1371/journal.pone.0244569 (PMC7806144; doi:10.1371/journal.pone.0244569)
Supplement: S6 Table — (DOCX) [file pone.0244569.s006.docx]

S6 Table. Results from the Kruskall-Wallis tests comparing SWL and affect balance between the study sites.

| Sites compared | SWL | | Affect balance | |
| --- | --- | --- | --- | --- |
|  | Chi-square | p | Chi-square | p |
| Roviana - Gizo | 0.56 | 0.454 | 0.23 | 0.631 |
| Gizo - Nijhum Dwip | 9.39 | 0.0022 | 37.98 | 7.2·10^-10^ |
| Nijhum Dwip - Chittagong | 23.57 | 1.2·10^-06^ | 4.76 | 0.029 |
| Roviana - Chittagong | 21.99 | 2.7·10^-06^ | 20.34 | 6.5·10^-06^ |
