# Supplementary material for: Happy without money: Minimally monetized societies can exhibit high subjective well-being
Source: PLoS One. 2021 Jan 13;16(1):e0244569. doi: 10.1371/journal.pone.0244569 (PMC7806144; doi:10.1371/journal.pone.0244569)
Supplement: S1 File — (DOCX) [file pone.0244569.s010.docx]

S1 File- Interview questions

Participant ID: ______________ Date: ______________________

Location: __________________ Interviewer: _________________

**Satisfaction with life**

All things considered, how satisfied are you with your life as a whole these days? Use a 0 to 10 scale, where 0 is dissatisfied and 10 is satisfied:

0 1 2 3 4 5 6 7 8 9 10

**Sociodemographics & personal background**

- 1. Male / Female
  2. Age? _____ years old
  3. What is your civil status?

Single civil partnership married divorced widow

- 1. Number of children? _____

Number of children living with you: _____

- 1. Main occupation: __________________________________
  2. Born in this village (lived here all your life)? YES / NO
  3. Ethnicity: ______________________________________

**Affect balance questions**

Now, please think about yesterday, from the morning until the end of the day. Think about where you were, what you were doing, who you were with, and how you felt.

Did you experience anger during a lot of the day yesterday? YES NO

Did you experience enjoyment during a lot of the day yesterday? YES NO

Did you experience happiness during a lot of the day yesterday? YES NO

Did you experience sadness during a lot of the day yesterday? YES NO

Did you experience stress during a lot of the day yesterday? YES NO

Did you experience worry during a lot of the day yesterday? YES NO

Did you learn or do something interesting yesterday? YES NO

Did you smile or laugh a lot yesterday? YES NO

Were you treated with respect all day yesterday? YES NO

Would you like to have more days just like yesterday? YES NO

Did you feel healthy yesterday? YES NO

**Income**

How much money does your household make? (introduce 4 relevant brackets:)

 0 – 500 SBD/month  500 – 2000 SBD/month

 2000 – 4000 SBD/month  4000 – 10000 SBD/month

**Monetization index**

What percentage of what you fish is sold?

 0% 25% 50% 75% 100%

How much of your food do you buy from a shop?

 0% 25% 50% 75% 100%

**ESM**

What are you feeling right now? Do you feel any of these emotions?

- 1. Satisfied
  2. Happy
  3. Smiling
  4. Fine, well
  5. Scared
  6. Worried
  7. Not good at all
  8. Angry
  9. Ill, sick
  10. Anxious to smoke
  11. Hungry
  12. Thirsty

**Sources of happiness**

What makes you happy? Please list the three things that make your happy, in order of importance.
